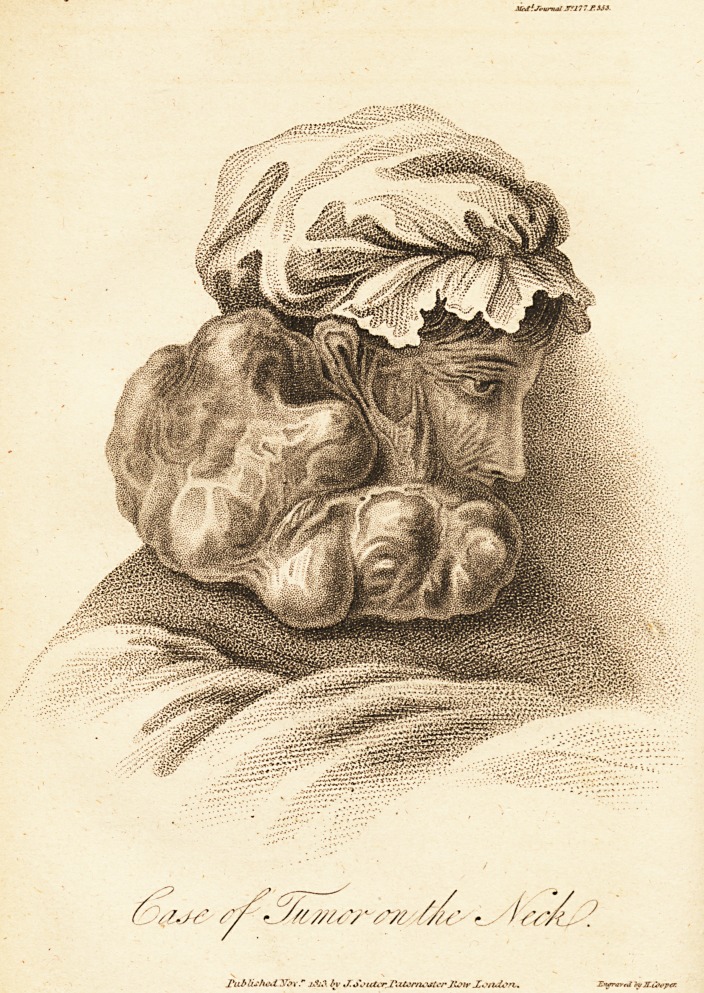# Mr. Atkinson's Case of Tumor on the Neck

**Published:** 1813-11

**Authors:** James Atkinson

**Affiliations:** Surgeon to His Royal Highness the Duke of York, Senior Surgeon to the York County Hospital and Dispensary


*Case of Tumor on the Neck.*

Published Novr. 1813 by J. Souter, Paternoster Row London. Engraved by H. Cooper.

THE
### Medical and Physical Journal.

5 OF VOL. XXX.] NOVEMBER, 1813. [NO. 177.

"For many fortunate discoveries in medicine, and for the detection of nume-
"rous errors, the world is indebted to the rapid circulation of Monthly
"Journals; and there never existed any work to which the faculty in
"EUROPE and AMERICA were under deeper obligations than to the Me-
"dical and Physical Journal of London, now forming a long, but an
"invaluable series."—RUSH.

### *To the Editors of the Medical and Physical Journal.*

(With a Plate.)

GENTLEMEN,

THE history of the annexed case is as follows. The subject of it was a middle-aged widow, and mother of several children.

Above twenty years ago, a tumor arose the size of a small pea, without any apparent cause, below the ear. The increase was slow and gradual, confining its dimensions for ten years to one small point. After that period other swellings commenced, until the size of the whole became such as is represented by the drawing. The weight and consequent pressure of the tumor produced some occasional vertigo, which was the sole inconvenience to the patient.

This case became unusually interesting, inasmuch as it was the opinion of a great number of respectable physicians and surgeons who had seen it, that an operation, should it ever be deemed practicable, would be attended with imminent danger; and the most unfavorable prognostic had been given by several others of the inevitable result of an operation.

For my own part, whatever apprehensions I might have as an operator, from the situation, condition, and circumstances of the case, yet, desperate as it was, my own opinion inclined decidedly for the operation. It was considered, in consultation, as practicable, however hazardous, and it was determined that if the patient chose to undergo the operation, and I had no objection to undertake it, that opinion should be my sanction.

The poor woman, in contemplating the situation and probable result of her case, as represented to her by the consulting gentlemen, evinced a firm and sensible mind.

Having a numerous family to rear under narrow circumstances, she had met and borne the inconvenience and difficulty of her malady with great fortitude. After two and twenty years' industrious exertions, she had managed to bring bring up her children. They were placed and settled in safety: of course she found herself at liberty to consult her own feelings, and, if necessary, to abide by any risque in relieving them. Whatever expedient she might choose to try, now solely implicated herself. With this view she sought advice, and the operation being adjudged indispensable, she submitted to it patiently.'

This was at any rate an awful decision, and it may be presumed that to confront the terror of an approaching dangerous expedient, in the face of numerous unfavorable forebodings, and of as numerous friends and visitors, would require something superadded to ordinary courage; for the size and appearance of the tumor rendered the patient an object both of opinion and of curiosity.

The operation was, of course, painful in proportion to the large extent of incised surface. Care was taken in performing it to preserve as much skin from the base of the tumor as should afford a covering to the wound. The incision was begun in that part of it by which the effusion of blood would least incommode the track of the scalpel. The inequalities consequent upon the lobular structure of the mass, had impressed some deep indentations within the neck; and the weight and bulk rendered it both unwieldy and inconvenient in operating.

On extirpating the tumor, a considerable arterial branch was unavoidably divided, which on the first jet of blood, and in that situation, had a formidable appearance. It was promptly restrained by pressure of the finger, until the operation was so far advanced as to make it convenient to secure it. A ligature was applied before the base of the tumor was separated, to prevent the occurrence of hemorrhage.

The tumor being completely removed, the carotid artery was observable in its course up the neck, exposed and pulsating. The large corresponding jugular vein presented also its broad, thinner, and less conspicuous coat. This became an object of notice only when certain compressions from motions of the neck allowed it to appear full, or to be emptied.

The incisions were obliged to be carried in two or three angular directions on the neck and cheek, yet they formed eventually a compact cicatrix; and the parts being carefully attended to during the healing process, left no very particular deformity. They were united and well in five or six weeks after the operation. The mouth, however, was rather disfigured from a certain unavoidable division of the nerves, and of course from a corresponding abatement of their influence upon the muscles.

The

The weight of the tumor was three pounds and nine ounces.

The patient had not any material bad symptoms during the cure.

The substance of the tumor consisted of a condensed smooth firm structure on the outermost lobes; and of a more pulpy, darker, greasy mass in the middle ones.

I have the honor to be, &c.

JAMES ATKINSON,

*Surgeon to His Royal Highness the Duke of York, Senior Surgeon to the York County Hospital and Dispensary.*

---

## Figures and Tables

**Figure f1:**